# A bearing fault diagnosis method for hydrodynamic transmissions integrating few-shot learning and transfer learning

**DOI:** 10.1038/s41598-025-04543-x

**Published:** 2025-05-30

**Authors:** Dong Sun, Xudong Yang, Hai Yang

**Affiliations:** 1 School of Mechanical Engineering, Guizhou University, Guiyang, 550025 China; 2 School of Mechanical Engineering, Guizhou Institute of Technology, Guiyang, 550025 China

**Keywords:** Hydrodynamic transmission, Bearing fault diagnosis, Few-shot learning, Transfer learning, Attention mechanism, Mechanical engineering, Mathematics and computing

## Abstract

To address the insufficient generalization capability of bearing fault diagnosis models caused by scarce vibration data from high-power hydrodynamic transmission testbeds, this study proposes a diagnostic method integrating deep few-shot learning with transfer learning. First, a Siamese Wide Convolutional Neural Network (Siamese-WDCNN) is constructed based on public bearing datasets to extract essential features of vibration signals through few-shot contrastive learning. Second, we introduce a transfer learning strategy to address cross-condition generalization challenges. This approach adapts pre-trained model parameters from the CWRU dataset to real industrial hydrodynamic transmission data. We then fine-tune the model using limited target-domain samples to optimize performance. Experiments evaluating the generalization capability under variable operating conditions compare diagnostic performance across SVM, WDCNN, WDCNN + TL, FSL + TL, and FSL + TL + AM methods. Results demonstrate that FSL + TL achieves an accuracy of 85.30% under mixed operating conditions. Further optimization by incorporating an attention mechanism (FSL + TL + AM) elevates accuracy to 88.75%, effectively enhancing the generalization capability of the bearing fault diagnosis model. This validates the engineering practicality of the proposed method and explores a viable pathway for industrial equipment health monitoring.

## Introduction

With the rapid advancement of industrial equipment toward high-power development, hydrodynamic transmissions, as core components of heavy-duty mechanical transmission systems, directly determine the operational reliability affecting production safety and economic efficiency in fields including construction machinery, mining equipment, and rail transportation. Bearings, serving as critical components enduring dynamic loads in hydrodynamic transmissions, are consistently subjected to high-speed, heavy-load, and variable operating conditions. These conditions predispose them to localized failures caused by abnormal assembly, fatigue wear, lubrication failure, or foreign particle intrusion, potentially triggering cascading equipment failures or safety incidents. Vibration monitoring and fault diagnosis technologies have garnered significant attention in equipment health management. Tiboni et al.^1^ systematically reviewed vibration-based condition monitoring methods, highlighting the integration of artificial intelligence and big data technologies as a future trend, though without in-depth exploration of applicability disparities across mechanical systems. Conventional fault diagnosis primarily relies on vibration signal analysis techniques^2^. These methods employ time-frequency domain feature extraction combined with classifiers such as support vector machines (SVM)^3–5^ and random forests for fault identification^6–8^. However, these methods heavily depend on expert-engineered features and exhibit limited adaptability to noise interference and operational condition variations. Consequently, they struggle to meet real-time diagnostic demands in complex industrial scenarios^9–11^.

Recent years have witnessed significant advancements in deep learning technologies for rotating machinery and industrial system fault diagnosis. Zhang et al.^12^ conducted comparative analyses revealing that deep learning algorithms exhibit a 33% lower false detection rate than traditional machine learning (ML) methods on the Case Western Reserve University (CWRU) bearing dataset. Huang et al.^13^ pioneered the integration of Convolutional Neural Network (CNN)and Long Short-Term Memory Network (LSTM) to construct a dual-modal network, incorporating sliding window techniques to capture fault-related temporal delay features, which reduced noise sensitivity in TE process diagnostics by 40%. Tama et al.^14^ posited that graph neural networks may resolve challenges in modeling the topological structure of vibration signals. Zhang et al.^15^ proposed converting 1D vibration signals into 2D images and leveraging Gated Recurrent Unit (GRU) to extract temporal features, achieving an 8.6% accuracy improvement under noisy conditions. Xie et al.^16^ implemented multi-sensor data fusion into RGB images, attaining 99.12% accuracy with a residual CNN model. Lei et al.^17^ delineated a three-generation technological roadmap (traditional ML → deep learning → transfer learning), identifying context-aware diagnostics as an emerging trend. Souza et al.^18^ validated the feasibility of low-cost monitoring in Industry 4.0 scenarios using single-sensor CNN models, though their work omitted performance degradation under 15% high-speed extreme operating conditions. Khan et al. ^19^systematically reviewed traditional statistical and deep learning methods for motor bearing diagnostics, emphasizing feature space mapping optimization as a critical challenge for enhancing algorithmic generalization. Jin et al. ^20^addressed fault prediction challenges in slewing bearings by integrating industrial case libraries with multi-physics monitoring data, proposing a dynamic risk scoring model that provides actionable solutions for yaw/pitch bearing health management. He et al^21^.proposed the Physics-Informed Wavelet Domain Adaptation Network (WIDAN), which integrates wavelet knowledge into convolutional layers to enhance cross-machine diagnostic performance. Li et al.^22^ introduced the Enhanced Kolmogorov-Arnold network with residual filtering(CLKAN-RF) Framework for anomaly detection in unmanned aerial vehicles (UAV) sensors, combining multiple networks and filtering mechanisms to improve detection accuracy. Wang et al. ^23^developed the Enhanced Transformer with Asymmetric Loss Function (ETALF) method for few-shot fault diagnosis with noisy labels, leveraging Transformer self-attention mechanisms and asymmetric loss functions to enhance model robustness. Li et al.^24^ designed an Energy-Driven Graph Neural OOD (EGN-OOD) Detector for out-of-distribution (OOD) fault analysis in construction machinery systems, significantly improving diagnostic precision and generalization capability. In summary, deep learning methodologies are progressively supplanting conventional signal processing techniques as the diagnostic core, with data dimensions evolving from single time-series to multi-source heterogeneous fusion, while model architectures increasingly adopt hybrid configurations.

However, existing deep learning models typically require extensive annotated training data, whereas real industrial scenarios often suffer from scarce and imbalanced fault sample distributions, leading to model overfitting under few-shot conditions. To address the challenge of data scarcity in industrial equipment fault diagnosis, Zero-Shot Learning (ZSL) and Few-Shot Learning (FSL) provide innovative solutions. Zero-shot diagnosis achieves the identification of unseen fault types through semantic attribute transfer or cross-domain feature alignment. Yang et al.^25^ proposed the Attribute Description Transfer based model for Zero-Shot Intelligent Diagnosis (ADT-ZSID) model based on attribute description transfer, which defines universal fault attributes to construct attribute classifiers and evaluates similarity for new fault diagnosis in high-voltage circuit breakers. Cai et al. ^26^further designed a multi-attribute learning model that utilizes shared multi-class attribute classifiers for cross-device diagnosis. To enhance cross-domain generalization, Ren et al. ^27^proposed the Meta Evolver framework with tensor prototype alignment, improving adaptability to unseen domains through uncertainty-aware dual meta-domain prototype learning. Few-shot diagnosis enhances model adaptability through metric learning and meta-learning. Zheng et al.^28^ introduced a Siamese Network (SN)-based method for bearing fault diagnosis, classifying samples via pairwise similarity. While it outperforms traditional models, its dependency on input pairing increases computational costs. Wang et al. ^29^proposed a Brain-Inspired Meta-Learning (BIML) strategy with Spiking Neural Networks (SNNs), demonstrating strong performance under few-shot conditions. Vu et al. ^30^developed a dual-branch model integrating Cross-Attention Transformers and Mahalanobis distance metrics, achieving improved diagnostic accuracy through multi-scale feature extraction and model ensemble in data-limited scenarios. Zhang et al.^31^ proposed a Model-Agnostic Meta-Learning (MAML)-based framework, achieving a 25% accuracy improvement over Siamese network benchmarks in artificial fault generalization tasks. It relies on task homogeneity, struggling with cross-domain generalization. Lin et al.^32^ developed a Generalized MAML (GMAML) with multi-kernel channel attention encoders, attaining an average precision of 97.6% in heterogeneous signal-driven cross-condition diagnostics, with its flexible weight adaptation mechanism accelerating new task adaptation by 40%. It incurs high computational costs and requires abundant labeled data. Li et al.^33^ innovatively integrated Spiking Neural Networks (SNN) with meta-contrastive learning, achieving 92.8% fine-grained diagnostic accuracy under heterogeneous fault categories. Wang et al.^34^ introduced the Few-Shot Meta Metric Model (FSM3) model combining supervised learning with metric meta-learning, demonstrating an 18% higher diagnostic accuracy than baseline methods in 5-shot scenarios. It lacks explicit domain alignment for industrial distribution shifts. Shao et al.^35^ optimized inner-loop residual networks using task-supervised Almost No Inner Loop (ANIL) algorithms, improving feature reuse efficiency by 31% in cross-domain bearing fault diagnosis. Ren et al.^36^ developed a Capsule Autoencoder (CaAE), reducing fault sample requirements from 100 + to 15 instances while cutting training time by 58%. Cheng et al.^37^ proposed a Multimodal Few-Shot Learning (MMFSL) framework that elevates industrial bearing diagnostic accuracy from 80.9 to 97.5% through joint temporal and image data generation. These studies focus on data scarcity scenarios, with deep learning-based feature extraction as the technical foundation. Experimental validations primarily rely on standard industrial datasets (e.g., the CWRU bearing dataset). Current FSL methods predominantly focus on single-device or fixed-condition scenarios, inadequately addressing cross-device and cross-condition domain shifts, However, challenges remain, such as limited model generalization capability and adaptability to complex operational conditions, thereby limiting their generalization capabilities in real-world industrial environments.

In the context of cross-condition diagnostics, Transfer Learning (TL) leverages knowledge from source-domain pre-trained models to provide prior feature representations for target domains, demonstrating unique advantages in cross-domain, few-shot, and partial domain adaptation scenarios. Zhao et al.^38^ established a comprehensive taxonomy for Unsupervised Transfer Learning (UDTL), revealing the dominant role of feature transferability in model performance. Chen et al.^39^ proposed a novel tripartite classification system based on label/machine/fault dimensions, identifying digital twin-edge computing integration as the next-generation technological core. Li et al.^40^ achieved a 23% average cross-condition accuracy improvement in gearbox fault diagnostics through conditional data alignment and unsupervised predictive consistency schemes. Deng et al.^41^ innovatively designed a Dual-Attention Generative Adversarial Network (DA-GAN) with hierarchical domain-sample attention to filter shared feature spaces, attaining 96.7% diagnostic accuracy in partial bearing fault transfer with 35% higher computational efficiency than conventional adversarial networks. Han et al.^42^ introduced a Paired Domain Adaptation(PDA) framework, eliminating negative transfer effects via single-sample independent domain matching and achieving 89.5% accuracy in cross-machine diagnostics. Li et al.^43^ developed a two-stage adversarial network combined with convolutional autoencoders for novel fault detection, attaining an F1-score of 0.91 in multi-gear compound fault scenarios.

This study addresses three core challenges in hydrodynamic transmission fault diagnosis:

1. Vibration signals from hydrodynamic transmissions are highly susceptible to interference from variable operating conditions. This makes conventional feature extraction methods inadequate for consistently capturing fault-sensitive features;
2. The target-domain hydrodynamic transmission system suffers from scarce fault samples. Additionally, significant structural and signal characteristic differences exist between source-domain public bearing datasets (e.g., CWRU) and the target domain. These factors make single-modality few-shot or transfer learning approaches insufficient to balance data efficiency with cross-domain generalization;
3. Industrial applications require diagnostic models with both high robustness and low computational overhead. Complex network architectures often struggle to satisfy these requirements due to real-time processing constraints.

To address these issues, this study proposes a bearing fault diagnosis framework (FSL + TL) that integrates deep few-shot learning with transfer learning, enabling efficient diagnosis under “few-shot + cross-condition” scenarios. First, a Siamese Wide Convolutional Neural Network (Siamese-WDCNN) is constructed using public datasets (CWRU), optimized through a few-shot contrastive learning strategy to enhance intra-class compactness and inter-class separability of fault features. Second, a transfer learning mechanism is introduced, where pre-trained source-domain models are adapted to the hydrodynamic transmission target domain by freezing front-end convolutional layers and fine-tuning classification layers, thereby minimizing domain distribution discrepancies. Finally, experiments under cold-start and imbalanced fault sample conditions are designed to validate the model’s robustness and generalization in real industrial scenarios.

While our method utilizes existing techniques, its novelty lies not in their simplistic combination but in the tailored architectural design and learning strategy specifically optimized for real-world hydrodynamic transmission fault diagnosis. Compared to existing methods, the contributions of this work are as follows:

We propose a hybrid FSL + TL framework with attention-enhanced discriminative learning, combining parameter-selective transfer and multi-scale alignment to address “few-shot + cross -condition” challenges. The framework dynamically prioritizes fault-sensitive frequency bands and ensures robust feature extraction under complex industrial scenarios, effectively enhances diagnostic generalization capability.

1. To address both cold-start few-shot conditions and imbalanced fault distributions in hydrodynamic transmissions, the framework validated on a real-world testbed, demonstrating practical utility beyond theoretical combinations of existing methods, providing a generalizable framework for industrial few-shot fault diagnosis.
2. Next, this paper is structured as follows: Sect. 2 reviews relevant theories and technologies, covering few-shot learning, transfer learning, and attention mechanisms; Sect. 3 details the design of the integrated few-shot and transfer learning diagnostic model, including the Siamese-WDCNN architecture and transfer fine-tuning strategies; Sect. 4 designs comparative experiments, including dataset partitioning and evaluation protocols; Sect. 5 validates the engineering practicality of the method using hydrodynamic transmission testbed data; Conclusions and future directions are discussed in Sect. 6.

## Materials and methods

### Few-shot learning (FSL)

Few-shot learning (FSL) is a machine learning methodology aimed at rapidly adapting to new tasks or categories using minimal annotated samples. Its core lies in constructing effective feature representations and metric mechanisms to prevent overfitting induced by data scarcity. This field primarily encompasses two main approaches: metric learning and meta-learning. In meta-learning, models enhance adaptability to novel tasks by learning how to learn, with the model-agnostic meta-learning (MAML) framework being the most representative method. MAML’s fundamental principle involves identifying an optimal model initialization through multi-task training, enabling rapid performance improvement on new tasks via minimal gradient updates. Specifically, for each task ${T_i}$, the inner-loop update rule is expressed as Eq. (1):

1 $${\theta ^{\prime}_i}=\theta - \alpha {\nabla _\theta }{L_{{T_i}}}(\theta )$$

where$\theta$denotes the meta-initialized parameters, $\alpha$represents the inner-loop learning rate, and ${L_{{T_i}}}(\theta )$ corresponds to the task-specific loss function.

The outer-loop objective of MAML is to optimize the initial parameters to minimize the aggregated loss across all tasks after inner-loop updates. The optimization objective is formulated as Eq. (2):

2 $${\hbox{min} _\theta }\sum\limits_{{{T_i}\sim p(T)}} {{L_{{T_i}}}} ({\theta ^{\prime}_i})=\sum\limits_{{{T_i}\sim p(T)}} {{L_{{T_i}}}} \left( {\theta - \alpha {\nabla _\theta }{L_{{T_i}}}(\theta )} \right)$$

Metric Learning constructs a feature embedding space where intra-class samples are clustered closely and inter-class samples are separated widely. The Siamese Neural Network, a widely used framework in metric learning, comprises two parameter-sharing subnetworks that classify input sample pairs by measuring their similarity. The contrastive loss function is defined as Eq. (3):

3 $$L=\frac{1}{{2 N}}\sum\limits_{{i=1}}^{N} {\left[ {{y_i}d_{i}^{2}+(1 - {y_i})\hbox{max} {{(0,m - {d_i})}^2}} \right]}$$

where *N* is the number of sample pairs, ${y_i}$is the label (${y_i}=1$ if samples belong to the same class,${y_i}=0$ otherwise), ${d_i}=\left\| {f(x_{i}^{1}) - f(x_{i}^{2})} \right\|$is the euclidean distance between sample pairs in the embedding space, *m* is the margin hyperparameter controlling the minimum separation between inter-class samples.

### Transfer learning(TL)

Transfer Learning is a technique that improves target-domain task performance by leveraging knowledge acquired from a source domain. Its core idea is to extract shared features between source and target domains to reduce their distribution discrepancy, enabling models trained on the source domain to adapt effectively to the target domain. It is primarily applied to scenarios with scarce data or high annotation costs. Implementation methods include feature transfer, instance transfer, and model transfer.

Feature Transfer minimizes distribution differences by projecting source- and target-domain data into a shared feature space via a mapping function. The distribution discrepancy is typically quantified using Maximum Mean Discrepancy (MMD), defined as Eq. (4):

4 $${\mathcal{L}_{MMD}}({\mathcal{D}_s},{\mathcal{D}_t})=\left\| {\frac{1}{{{n_s}}}\sum\limits_{{i=1}}^{{{n_s}}} \phi (x_{i}^{s}) - \frac{1}{{{n_t}}}\sum\limits_{{j=1}}^{{{n_t}}} \phi (x_{j}^{t})} \right\|_{\mathcal{H}}^{2}$$

where${\mathcal{D}_s}$and${\mathcal{D}_t}$represent the source and target domain data distributions, ${n_s}$and ${n_t}$ are the number of samples from each domain, $x_{i}^{s}$and $x_{j}^{t}$ are feature representations from source and target domains respectively, $\phi ( \cdot )$is the mapping function to the Reproducing Kernel Hilbert Space (RKHS), and $|| \cdot ||_{\mathcal{H}}$ denotes the norm in the Hilbert space.

Domain-Adversarial Neural Networks (DANN) learn domain-invariant features by introducing a domain discriminator in an adversarial manner. The objective function comprises task-specific classification loss and domain adversarial loss, formulated as Eq. (5):

5 $${\hbox{min} _{G,F}}{L_y}(G,F) - \lambda {L_d}(G,D)$$

where *G* is the feature extractor, *F* is the task classifier, *D* is the domain discriminator, ${L_y}(G,F)$ is the classification loss on the source domain, ${L_d}(G,D)$ is the Domain adversarial loss, $\lambda$is the hyperparameter balancing the two losses.

Joint Distribution Adaptation (JDA) considers both marginal and conditional distribution discrepancies. Its objective function is given by Eq. (6):

6 $${\hbox{min} _F}\mid\mid {\mu _s} - {\mu _t}{\mid\mid ^2}+\sum\limits_{{c=1}}^{C} {\mid\mid \mu _{s}^{c} - \mu _{t}^{c}{\mid\mid ^2}}$$

where ${\mu _s}$and${\mu _t}$are the global means of source and target domain samples; $\mu _{s}^{c}$and $\mu _{t}^{c}$are the class-specific means; *C* is the total number of classes.

### Attention mechanism(AM)

Attention Mechanism captures critical information by adaptively weighting input components based on their importance. Its core idea utilizes similarity scores between queries (*Q*), keys (*K*), and values (*V*) to generate representative feature representations. The scaled dot-product attention is computed as Eq. (7):

7 $$Attention(Q,K,V)={\text{softmax}}\left( {\frac{{Q{K^T}}}{{\sqrt {{d_k}} }}} \right)V\frac{1}{2}$$

where $Q \in {{\mathbb{R}}^{n \times {d_k}}}$ denotes the query matrix, $K \in {{\mathbb{R}}^{m \times {d_k}}}$ is the key matrix, and $V \in {{\mathbb{R}}^{m \times {d_v}}}$ is the value matrix, ${d_k}$represents the dimension of the key vectors. The mechanism calculates the scaled dot product of *Q* and *K*, applies softmax normalization to generate attention weights, and subsequently computes a weighted sum with *V* to produce the final contextual representation.

Multi-Head Attention enhances feature representation by computing multiple attention heads in parallel, as shown in Eq. (8):

8 $$MultiHead(Q,K,V)={\text{Concat}}(hea{d_1},hea{d_2}, \ldots ,hea{d_h}){W^O}$$

Each head is computed as Eq. (9):

9 $$hea{d_i}={\text{Attention}}(QW_{i}^{Q},KW_{i}^{K},VW_{i}^{V})$$

where $W_{i}^{Q}$,$W_{i}^{K}$,$W_{i}^{V}$is the linear transformation matrices for the i-th head; ${W^O}$is the output transformation matrix.

The self-attention mechanism utilizes the input sequence itself as the queries (*Q*), keys (*K*), and values (*V*), enabling the capture of interdependencies among elements within the sequence. Its computational process is identical to that described in Eq. (7).

Additive Attention computes relevance scores via Eq. (10):

10 $${e_{ij}}={v^T}\tanh ({W_1}{q_i}+{W_2}{k_j})$$

Normalized attention weights are obtained using Eq. (11):

11 $${\alpha _{ij}}=\frac{{\exp ({e_{ij}})}}{{\sum\limits_{{j^{\prime}}} {\exp } ({e_{ij^{\prime}}})}}$$

The context vector is generated by a weighted sum of keys, as in Eq. (12):

12 $${c_i}=\sum\limits_{j} {{\alpha _{ij}}} {k_j}$$

where ${q_i}$and ${k_j}$are the query and key vectors; ${W_1}$and${W_2}$ are the linear transformation matrices; *v* is the projection vector.

## Model architecture

**Fig. 1:**
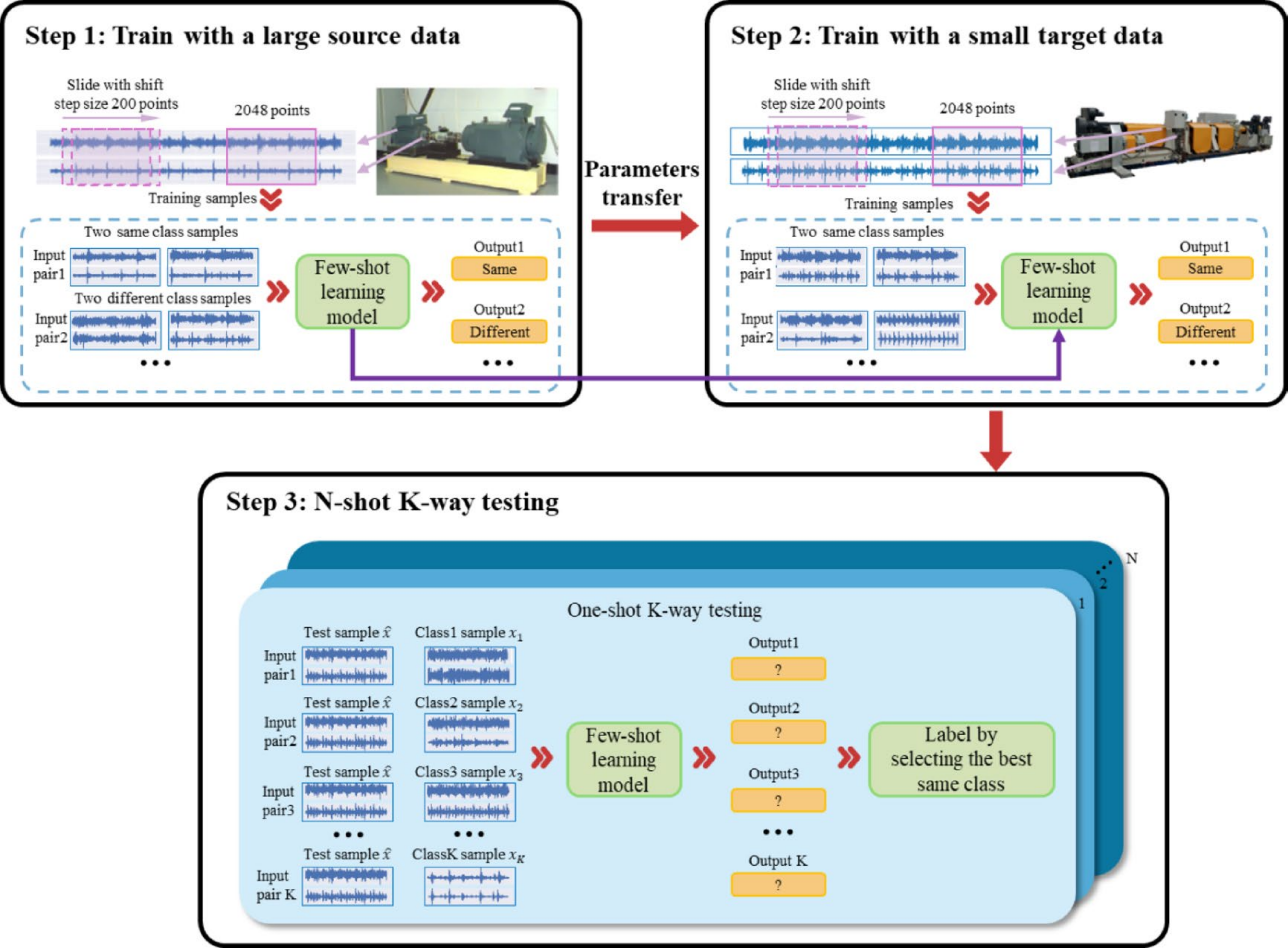
FSL + TL Model Architecture.

Under data-scarce conditions in target-domain hydrodynamic transmission testbeds, achieving high-accuracy bearing fault diagnosis constitutes the core challenge addressed in this study. To resolve this, we propose an FSL + TL method that synergistically integrates few-shot contrastive learning with transfer learning. This approach simultaneously leverages the Siamese network’s capability for effective feature learning with limited samples and exploits pre-trained model transfer to minimize distributional discrepancies between source and target domains, thereby achieving high generalization in cross-condition fault diagnosis. The model architecture comprises two key modules: (1) a Siamese Wide Convolutional Neural Network (Siamese-WDCNN) pre-trained on public datasets, and (2) a transfer fine-tuning module tailored for target-domain data, as illustrated in Fig. 1.

**Fig. 2:**
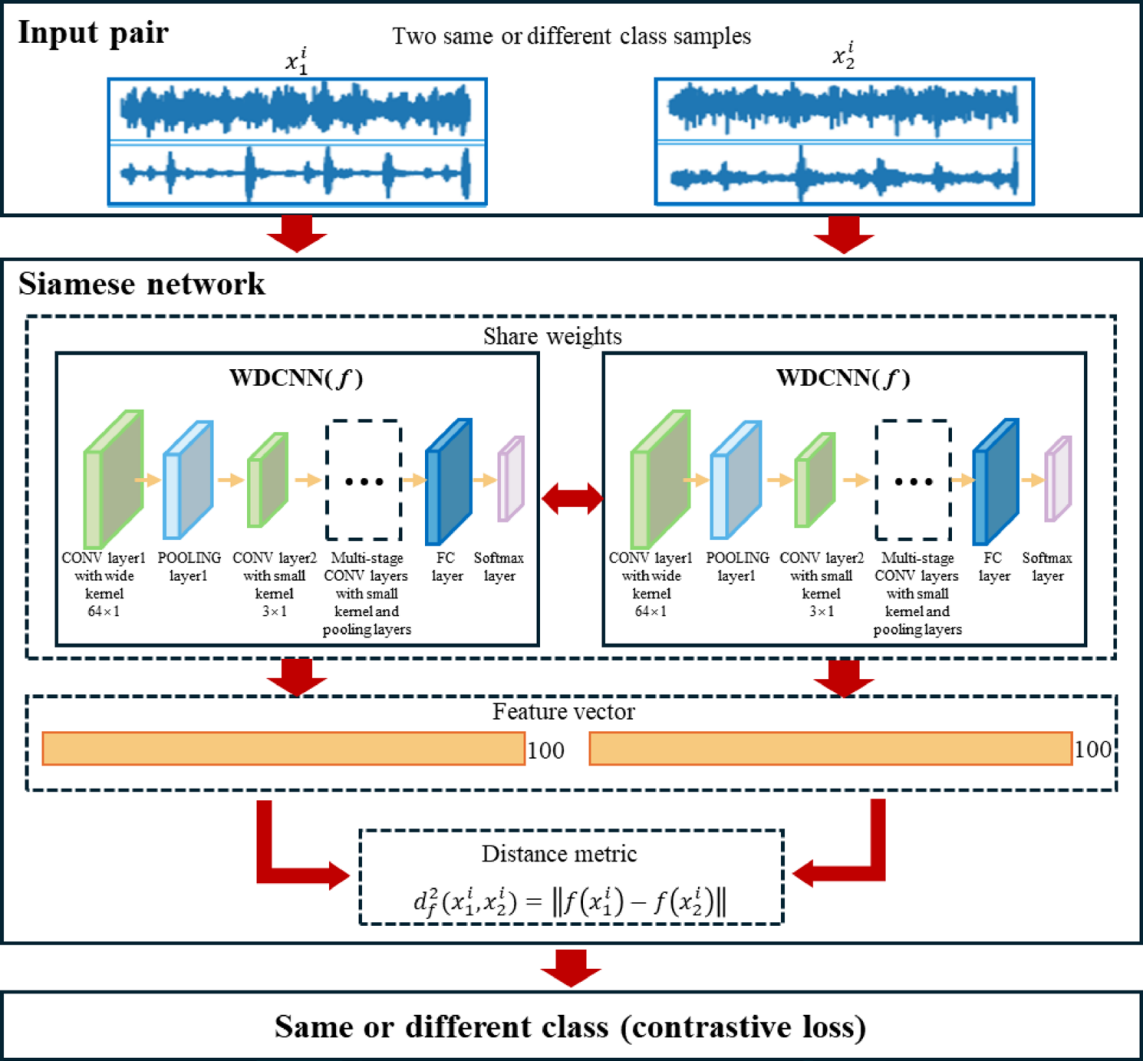
WDCNN-Based Few-Shot Learning Model.

During the pre-training phase, the Siamese network structure shown in Fig. 2 is adopted. Two weight-sharing WDCNN branches extract features from vibration signals^44^. The WDCNN architecture configuration, identical to that described in Table 1 and Reference^44^, employs a first-layer convolutional kernel of size 64 × 1 with a stride of 16 × 1. This wide-kernel filtering suppresses high-frequency noise (e.g., electromagnetic interference, mechanical impacts) while retaining low-frequency fault features such as bearing periodic impacts. Subsequent layers utilize 3 × 1 small kernels to refine local features and capture time-frequency details of fault signals. The contrastive loss function minimizes intra-class distances and maximizes inter-class separations in the embedding space, thereby enhancing discriminative capability for few-shot classification. For pre-training, the CWRU bearing dataset serves as source-domain data to construct few-shot tasks across fault categories. The Siamese-WDCNN is trained using the Adam optimizer to improve adaptability to operational condition variations.

**Table 1:** Few-Shot learning parameters of the WDCNN framework.

No.	Layer type	Kernel size/Stride	Kernel number	Output size (Widthx Depth)	Padding
1	Convolution1	64 × 1/16 × 1	16	128 × 16	same
2	Pooling1	2 × 1/2 × 1	16	64 × 16	valid
3	Convolution 2	3 × 1/1 × 1	32	64 × 32	same
4	Pooling 2	2 × 1/2 × 1	32	32 × 32	valid
5	Convolution 3	3 × 1/1 × 1	64	32 × 64	same
6	Pooling 3	2 × 1/2 × 1	64	16 × 64	valid
7	Convolution 4	3 × 1/1 × 1	64	16 × 64	same
8	Pooling 4	2 × 1/2 × 1	64	16 × 64	valid
9	Convolution 5	3 × 1/1 × 1	64	6 × 64	valid
10	Pooling 5	2 × 1/2 × 1	64	3 × 64	valid
11	Fully-connected	100	1	100 × 1	

During the transfer learning phase, the pre-trained Siamese network serves as the base model. By freezing the front-end convolutional layers that capture generic low-level features, only the fully connected layers and subsequent classification layers are fine-tuned to adapt to specific fault patterns in the hydrodynamic transmission target domain. To further reduce distribution discrepancies between source and target domains, a domain alignment loss term is introduced during fine-tuning, leveraging minimal labeled target-domain data for backpropagation updates.

The domain alignment loss is a critical component of our transfer learning strategy, designed to minimize the distribution discrepancy between source and target domains. We implement this loss using the Maximum Mean Discrepancy (MMD) metric, which measures the distance between the means of two probability distributions mapped to a Reproducing Kernel Hilbert Space (RKHS). The domain alignment loss is formulated as Eq. 4 in Sect. 2.2. In practice, we implement this using the kernel trick with a mixture of multiple RBF kernels:

13 $$k({x_i},{x_j})=\sum\limits_{{q=1}}^{Q} {\exp } \left( { - \frac{{\mid\mid {x_i} - {x_j}{\mid\mid ^2}}}{{2\sigma _{q}^{2}}}} \right)$$

where ${\sigma _q}$ represents different bandwidth parameters. In our implementation, we use $Q=5$ kernels with ${\sigma _q} \in \{ 0.01,0.1,1,10,100\}$ to capture similarities at multiple scales. The final loss function for our transfer learning phase combines the domain alignment loss with the task-specific classification loss:

14 $${\mathcal{L}_{total}}={\mathcal{L}_{cls}}+\lambda {\mathcal{D}_t}$$

where ${\mathcal{L}_{cls}}$ is the cross-entropy loss for fault classification, and $\lambda$ is a trade-off parameter set to 0.5 in our experiments based on validation performance.

The entire fine-tuning process employs early stopping and cross-validation techniques to prevent overfitting and ensure model stability. Upon training completion, the model is tested on target-domain data to validate its generalization capability and robustness across varying operating conditions.

The advantages of the proposed method are highlighted as follows:

1. The few-shot contrastive learning framework with the Siamese network effectively overcomes the limitations of traditional methods in extracting fault features under extremely limited data conditions;
2. The transfer learning strategy enables the reuse of generic fault features learned from the CWRU dataset for the hydrodynamic transmission target domain, achieving rapid adaptation through fine-tuning and efficient cross-domain knowledge utilization;
3. The domain alignment strategy significantly mitigates distribution shifts between source and target domains, thereby improving diagnostic accuracy under variable operating conditions;
4. The overall architecture balances robust feature extraction with classifier lightweighting, meeting real-time and low-computational demands in industrial settings, offering an efficient and robust solution for equipment fault diagnosis.

## Experimental design

### Dataset description

**Fig. 3:**
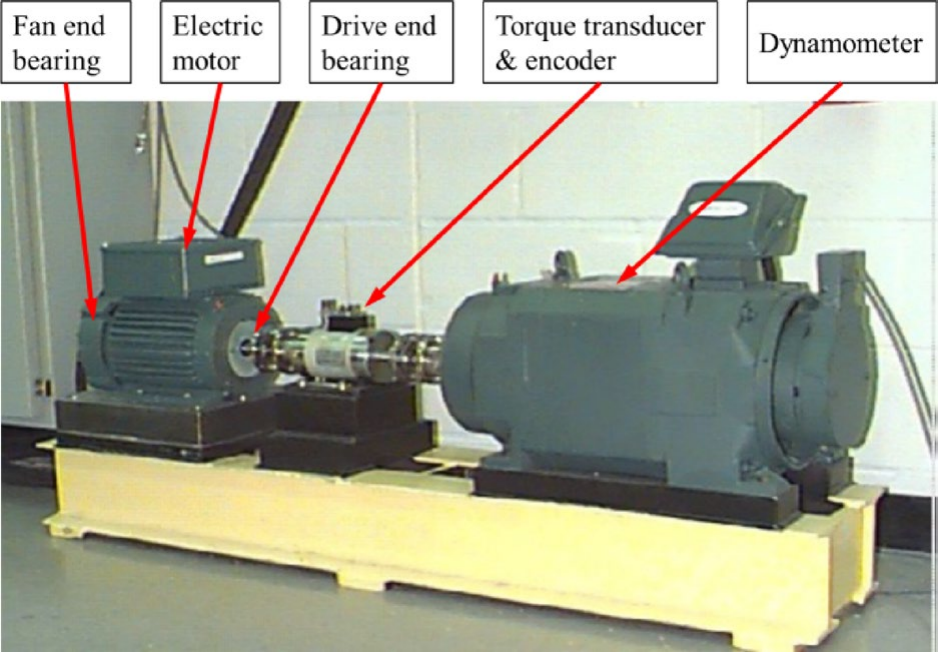
Bearing Fault Test Rig Used in CWRU Dataset.

The source domain data utilizes the 12 kHz drive-end bearing fault dataset from the publicly available Case Western Reserve University (CWRU) bearing fault database, as shown in Fig. 3. The dataset includes four bearing states: normal, ball fault, inner race fault, and outer race fault, with motor speeds of 1772 r/min, 1750 r/min, and 1730 r/min. Vibration signals are collected via accelerometers, and training samples are generated using a sliding window of length 2048 and step size 80. Each fault category contains 4500 training samples, totaling 18,000 samples.

**Fig. 4:**
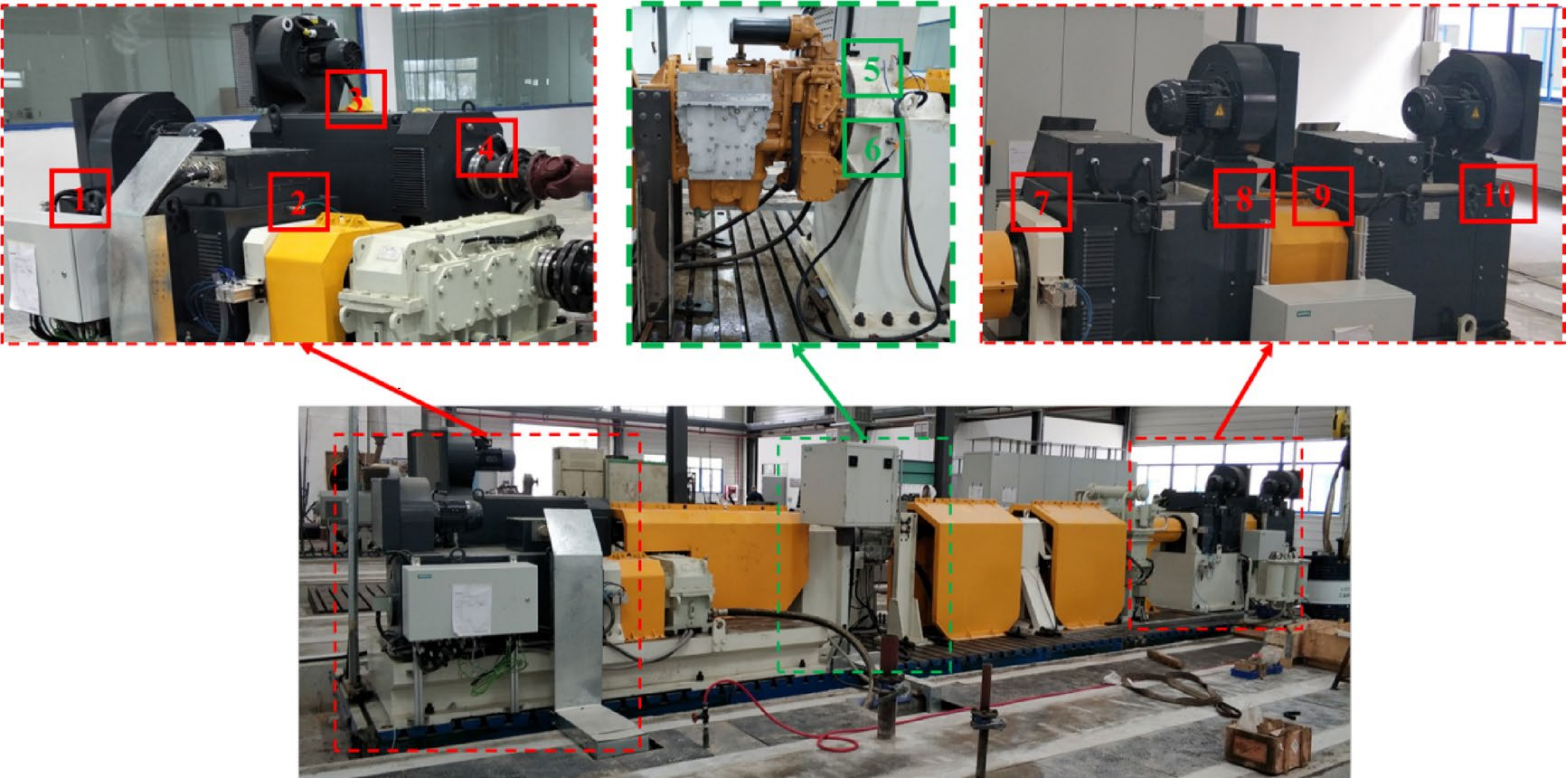
Vibration Sensor Layout on the Hydrodynamic Transmission Testbed.

The target domain data is collected from a high-power hydrodynamic transmission testbed independently developed by an industrial enterprise. The testbed integrates a vibration monitoring system with IFM accelerometers installed at the drive end of one drive motor, three load motors, fan ends, and transmission mounting brackets, as shown in Fig. 4. This study utilizes vibration data from sensors #5 and #6 mounted on the X- and Y-axes at the drive end of the hydrodynamic transmission. Bearing states similarly include normal, inner race fault, outer race fault, and ball fault, with signal lengths standardized to 2048 data points to align with source-domain specifications. Following test protocols, the drive motor operates at 400–2000 r/min. Training sets TR1, TR2, and TR3 collect 200 samples per fault category under three conditions (400 r/min, 1000 r/min, 1800 r/min). Test sets TS1, TS2, and TS3 include three novel operating conditions (800 r/min, 1200 r/min, 1500 r/min) and a mixed-condition TS1 + 2 + 3, with 30 samples per fault category, to evaluate cross-condition generalization.

The finalized source- and target-domain datasets are summarized in Table 2.

**Table 2:** Rolling bearing dataset specifications.

Fault Location		Normal	Inner Race	Ball	Outer Race	Speed(*r*/min)
Fault Labels		0	1	2	3	/
CWRU	Train	4500	4500	4500	4500	1772\1750\1730
TR1	Train	200	200	200	200	400
TR2	Train	200	200	200	200	1000
TR3	Train	200	200	200	200	1800
TS1	Test	30	30	30	30	800
TS2	Test	30	30	30	30	1200
TS3	Test	30	30	30	30	1500
TS1 + 2 + 3	Test	90	90	90	90	800\1200\1500

### Experimental design

This experiment is designed to address the cross-condition generalization challenge in hydrodynamic transmission bearing fault diagnosis, while accounting for two critical challenges: cold-start few-shot conditions and imbalanced fault samples. The specific experimental configurations are detailed in Table 3.

To address the cold-start scenario with extremely limited target-domain training data, the training set selects 20 samples per group from datasets TR1, TR2, and TR3, with 5 samples per fault label. The test sets (TS1, TS2, TS3, and mixed-condition TS1 + 2 + 3) are evaluated using the following methods: Traditional machine learning (SVM), Pure deep learning (WDCNN), Transfer learning-enhanced WDCNN + TL, Few-shot learning with Siamese networks (FSL) and the proposed FSL + TL method. This configuration validates the model’s rapid cross-condition adaptation capability under cold-start few-shot constraints.

For the imbalanced fault sample scenario, where certain fault categories have sufficient samples under specific operating conditions while others remain few-shot, the training set combines CWRU pre-trained models with full fine-tuning on TR1, TR2, and TR3. The test set (TS1 + 2 + 3) are evaluated using the baseline FSL + TL method and the enhanced FSL + TL + AM method (with attention mechanism).This assesses the model’s robustness and generalization under imbalanced data distributions.

**Table 3:** Experimental design.

Method	Description	Training Data	Testing Data	Targeted Testing Scenario
SVM	Support Vector Machine	20 samples per group from TR1, TR2, TR3 (5 samples per class)	TS1、TR2、TR3 TS1 + 2 + 3	Cold-start few-shot fault diagnosis model (extremely limited samples per class initially)
WDCNN	Wide Kernel Deep Convolutional Network	20 samples per group from TR1, TR2, TR3 (5 samples per class)	TS1、TR2、TR3 TS1 + 2 + 3	
FSL	Siamese Network Architecture (Metric Learning for Sample Pair Similarity)	20 samples per group from TR1, TR2, TR3 (5 samples per class)	TS1、TR2、TR3 TS1 + 2 + 3	
WDCNN + TL	Transfer Learning-Based Approach	CWRU model + 20 samples per group from TR1, TR2, TR3 (5 samples per class)	TS1、TR2、TR3 TS1 + 2 + 3	
FSL + TL (Few-Shot Fine-Tuning)	Siamese Network Pre-training + Target-Domain Few-Shot Fine-Tuning	CWRU model + 20 samples per group from TR1, TR2, TR3 (5 samples per class)	TS1、TR2、TR3 TS1 + 2 + 3	
FSL + TL (Full Fine-Tuning)	Siamese Network Pre-training + Moderate Target-Domain Fine-Tuning	CWRU model + Full fine-tuning on TR1, TR2, TR3	TS1 + 2 + 3	Imbalanced fault diagnosis (some faults have sufficient samples, others remain few-shot)
FSL + TL + AM	Integration of Attention Mechanism	CWRU model + Full fine-tuning on TR1, TR2, TR3	TS1 + 2 + 3	

### Experimental environment

In this study, a strictly controlled experimental environment is used to build the computational experimental platform, and the training system is constructed based on NVIDIA GeForce RTX 3060 graphics processor and Pytorch 1.40 deep learning framework. In terms of hyper-parameter optimization, the optimal configuration is determined by grid search.The batch size is fixed at 64 to balance the memory consumption and gradient stability. Smaller batch sizes (< 32) led to high variance in gradient updates and unstable training, while larger batch sizes (> 128) resulted in smoother but slower convergence without significant accuracy improvements. We employed different learning rates for the few-shot learning and transfer learning phases by pre-experiment. For the initial few-shot learning phase, we used 0.001 to ensure stable convergence of the Siamese network. During transfer learning, we adopted a lower learning rate 0.0005 for fine-tuning to prevent catastrophic forgetting of previously learned features. The margin parameter in the contrastive loss function controls the minimum distance between samples from different classes in the embedding space. We evaluated margins ranging from 0.5 to 2.5 and found that 1.5 provides the optimal balance between intra-class compactness and inter-class separability. Smaller margins (< 1.0) result in insufficient separation between fault categories, while larger margins (> 2.0) cause training instability and overfitting, particularly under few-shot conditions.The model training period is 200 epochs to ensure that the model fully converges while avoiding the risk of overfitting. In order to enhance the statistical reliability of the experimental results, the experiments were repeated five times independently under each identical condition, and the average value was finally taken as the index for assessing the performance.

## Testing and analysis

### Testing and analysis of few-shot fault diagnosis models under cold-start conditions

**Fig. 5:**
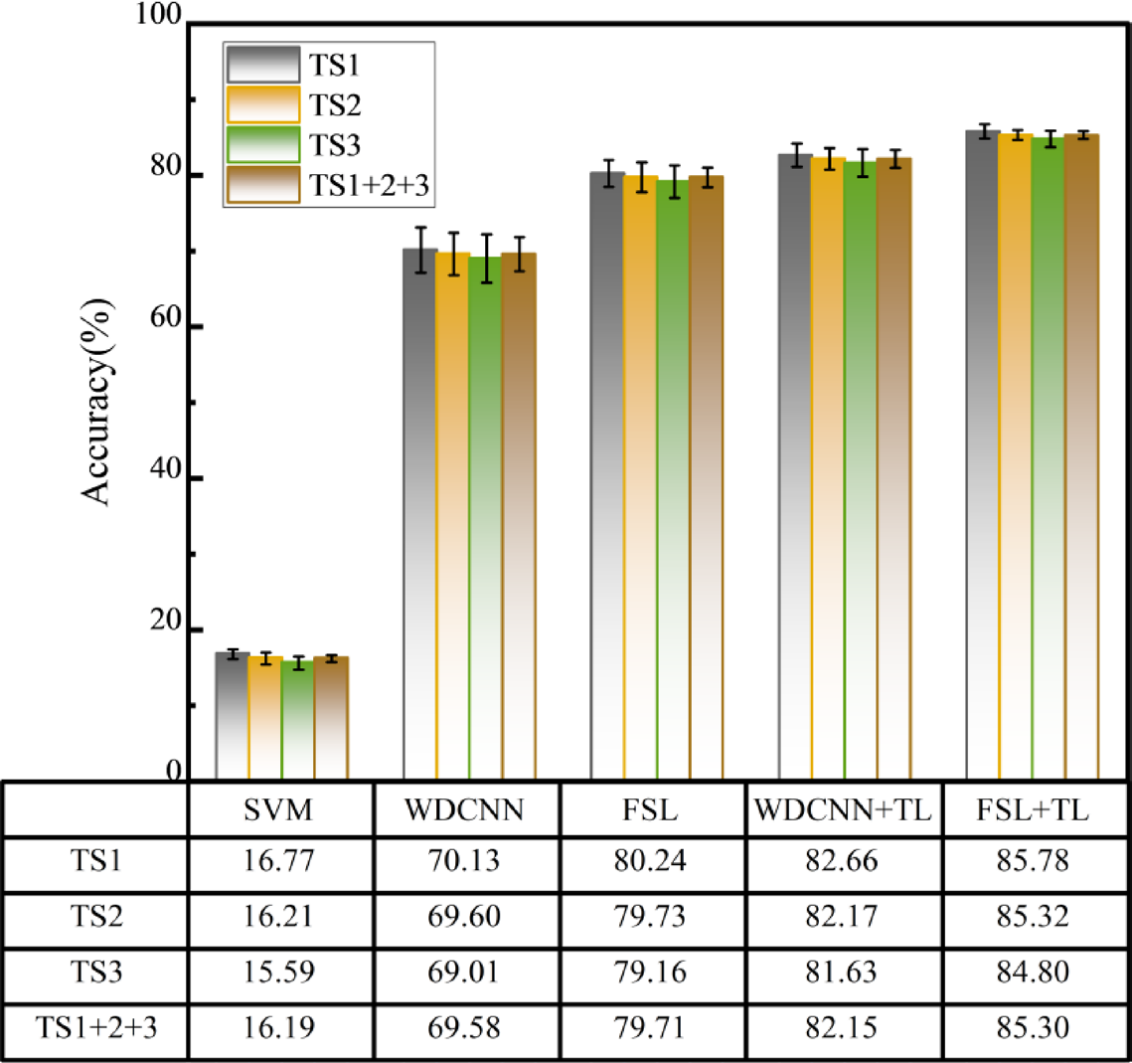
Diagnostic Results of Different Methods Under Cold-Start Conditions.

Following the experimental protocol, the target-domain test data are partitioned into four subsets: Single-condition TS1 (800 r/min, 30 samples per fault class, 120 total), Single-condition TS2 (1200 r/min, 30 samples per fault class, 120 total), Single-condition TS3 (1500 r/min, 30 samples per fault class, 120 total) and Mixed-condition TS1 + 2 + 3 (combined data from three conditions, 90 samples per fault class, 360 total).SVM, WDCNN, FSL, WDCNN + TL, and FSL + TL methods were comparatively analyzed. Diagnostic results are illustrated in Fig. 5.

The results demonstrate that traditional SVM achieves low accuracy across all conditions (16.77%, 16.21%, 15.59%, and 16.19%), indicating its inability to capture complex fault patterns under few-shot constraints. WDCNN exhibits improved performance (70.13%, 69.60%, 69.01%, and 69.58%), validating the advantage of end-to-end deep learning in automatic feature extraction, though overfitting and robustness issues persist under data scarcity. The FSL method, leveraging contrastive learning via Siamese networks, achieves 80.24%, 79.73%, 79.16%, and 79.71% accuracy, outperforming WDCNN by 10.13% in mixed conditions, thereby confirming the efficacy of few-shot learning in low-sample regimes. However, FSL alone suffers from cross-domain distribution bias, resulting in accuracy fluctuations across speed conditions. By integrating transfer learning (TL) to fine-tune pre-trained source-domain parameters, WDCNN + TL attains 82.66%, 82.17%, 81.63%, and 82.15% accuracy, surpassing WDCNN and FSL by 12.57% and 2.44%, respectively, in mixed conditions. The above experimental results show that SVM performs poorly due to its reliance on linear assumptions, failing to capture nonlinear fault patterns under few-shot conditions. WDCNN improves accuracy by learning hierarchical features from raw data, yet overfits with limited samples. FSL leverages contrastive learning to enhance feature separability but suffers from cross-domain bias. WDCNN + TL addresses this via transfer learning: fine-tuning preserves generic features while aligning domains, balancing data efficiency and generalization.

To fully exploit the efficacy of few-shot learning under low-sample conditions and the advantage of transfer learning in leveraging pre-trained model priors under data scarcity, the proposed FSL + TL method first pre-trains the Siamese network on the CWRU dataset, followed by fine-tuning with limited target-domain samples. Experimental results demonstrate that this method achieves accuracy rates of 85.78%, 85.32%, 84.80%, and 85.30% across all operating conditions, outperforming all comparative methods. Specifically, it surpasses the FSL method by 5.54%, 5.59%, and 5.64% in TS1, TS2, and TS3, respectively, and exceeds the TL method by 3.12%, 3.15%, and 3.17% in the same conditions. The superior performance of FSL + TL stems from its dual-phase knowledge integration: pre-training on the CWRU dataset captures universal fault patterns, while fine-tuning with limited target-domain data aligns domain-specific features. This hybrid approach mitigates cross-domain bias inherent in pure FSL and enhances data efficiency beyond standalone TL, achieving consistent accuracy gains by balancing generalization and adaptation.

**Fig. 6:**
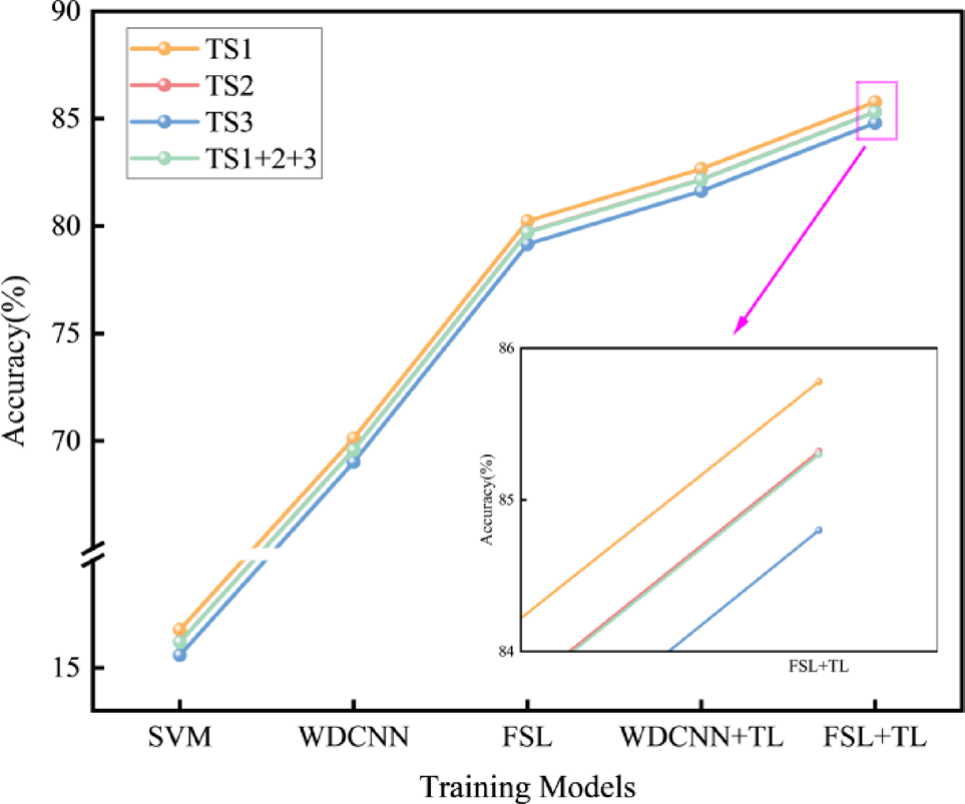
Influence of Rotational Speed on Diagnostic Accuracy.

Notably, TS1 (lowest speed) yields the highest accuracy, with slight declines observed at higher speeds (TS2, TS3), as shown in Fig. 6. This aligns with mechanical vibration principles, where low-speed conditions amplify fault-related features.

**Fig. 7:**
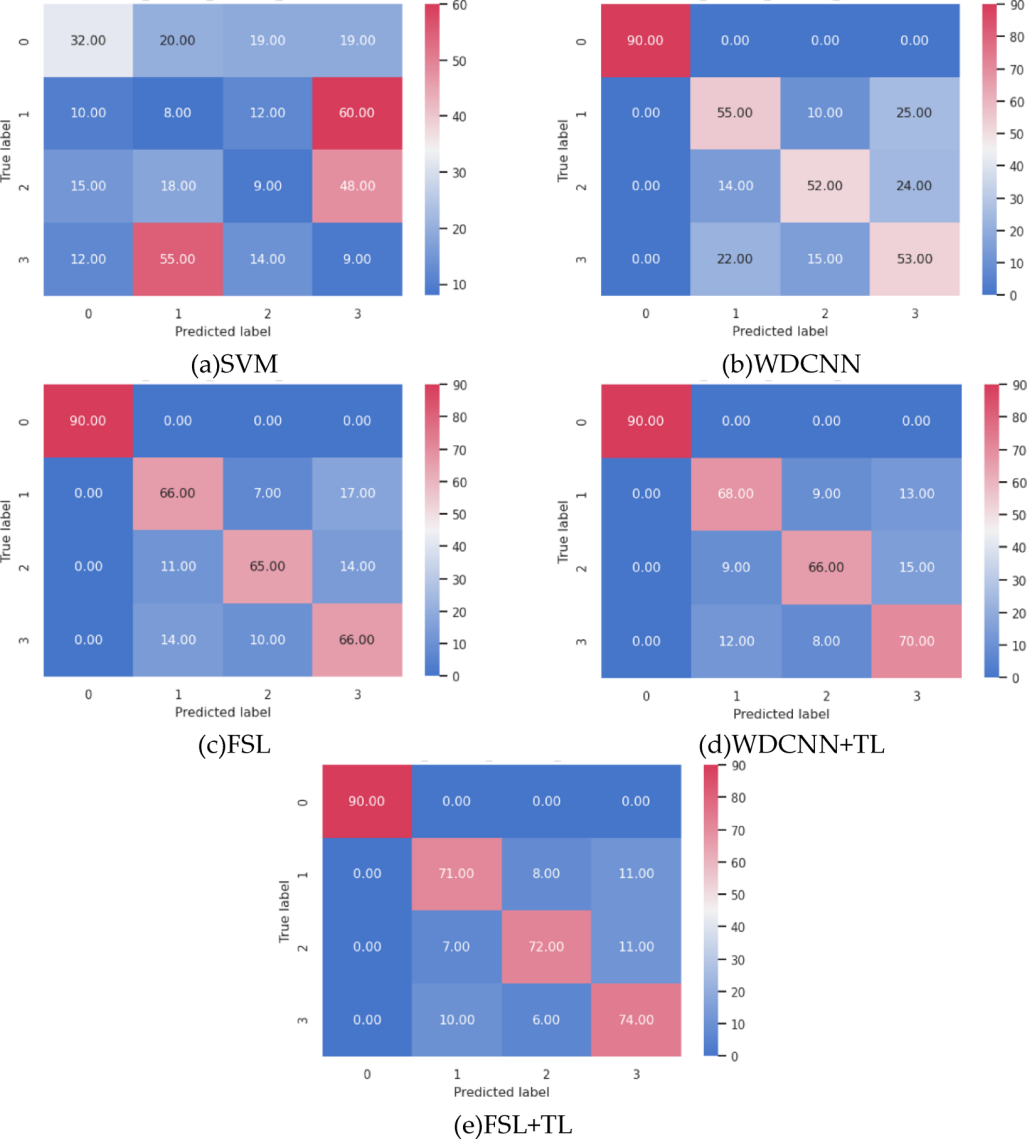
Confusion Matrices of Diagnostic Results.

For intuitive evaluation, Fig. 7 presents confusion matrices for all five methods under mixed conditions using 360 test samples.

Further evaluation metrics—precision, recall, F1-score, and macro-average—are quantified in Table 4.

**Table 4:** Evaluation metrics of comparative methods.

Method	Index	0	1	2	3	Macro-avg
SVM	Precision(%)	46.38	7.92	16.67	6.62	19.40
	Recall(%)	35.56	8.89	10.00	10.00	16.11
	F1-Score	0.4	0.08	0.12	0.08	0.17
WDCNN	Precision(%)	100	60.44	67.53	51.96	69.98
	Recall(%)	100	61.11	57.78	58.89	69.45
	F1-Score	1.00	0.61	0.62	0.55	0.70
FSL	Precision(%)	100	72.53	79.27	68.04	79.96
	Recall(%)	100	73.33	72.22	73.33	79.72
	F1-Score	1.00	0.73	0.76	0.71	0.80
WDCNN + TL	Precision(%)	100	76.40	79.52	71.43	81.84
	Recall(%)	100	75.56	73.33	77.78	81.67
	F1-Score	1.00	0.76	0.76	0.74	0.82
FSL + TL	Precision(%)	100	80.68	83.72	77.08	85.37
	Recall(%)	100	78.89	80.00	82.22	85.28
	F1-Score	1.00	0.80	0.82	0.80	0.85

Analysis of confusion matrices and evaluation metrics across methods reveals that SVM exhibits significantly lower performance in all metrics. Specifically, its prediction rate for the normal state (Class 0#) is merely 36%, with severe misclassification observed for inner race (Class 1#) and outer race (Class 3#) faults, indicating ineffective vibration signal feature extraction under cold-start few-shot conditions, leading to widespread fault misidentification. In contrast, WDCNN achieves 100% correct predictions for the normal state, with a macro-average F1-score of 0.7—4.1× higher than SVM. However, it still struggles to distinguish subtle fault features between inner and outer race faults under few-shot constraints. The FSL method substantially reduces inter-fault confusion, elevating the macro-average F1-score to 0.8. With transfer learning integration, WDCNN + TL and FSL + TL further improve macro-average F1-scores to 0.82 and 0.85, respectively, confirming TL’s efficacy in mitigating data scarcity. These findings demonstrate that the FSL + TL method, by synergizing few-shot learning and transfer learning, achieves optimal overall accuracy and minimizes inner/outer race fault misclassification. FSL employs contrastive learning to extract discriminative features from limited data, enhancing separability between subtle fault classes. Meanwhile, TL bridges domain gaps by fine-tuning pre-trained models on hydrodynamic transmission data, selectively preserving domain-invariant features while aligning target-specific characteristics.

Fault-class-specific analysis shows all methods perform best on Class 0#, while Classes 1# and 3# remain challenging, with peak F1-scores of 0.8. Class 2# exhibits consistently lower recall than precision, suggesting under-detection risks for this fault type.

### Testing and analysis of few-shot fault diagnosis models under imbalanced fault samples

**Fig. 8:**
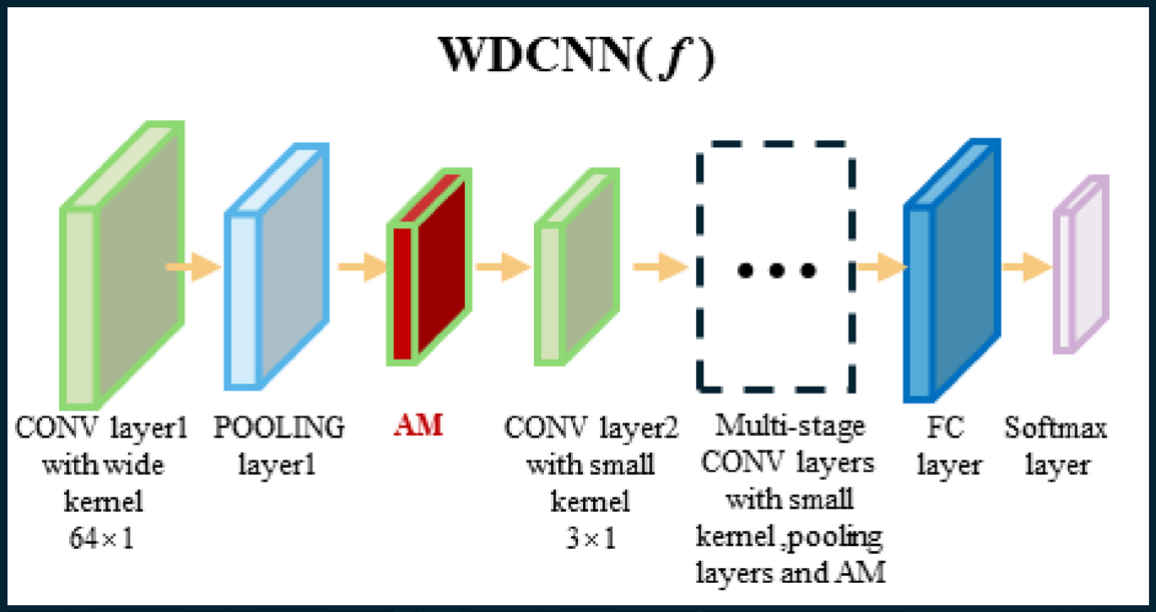
WDCNN with Integrated Attention Mechanism.

**Fig. 9:**
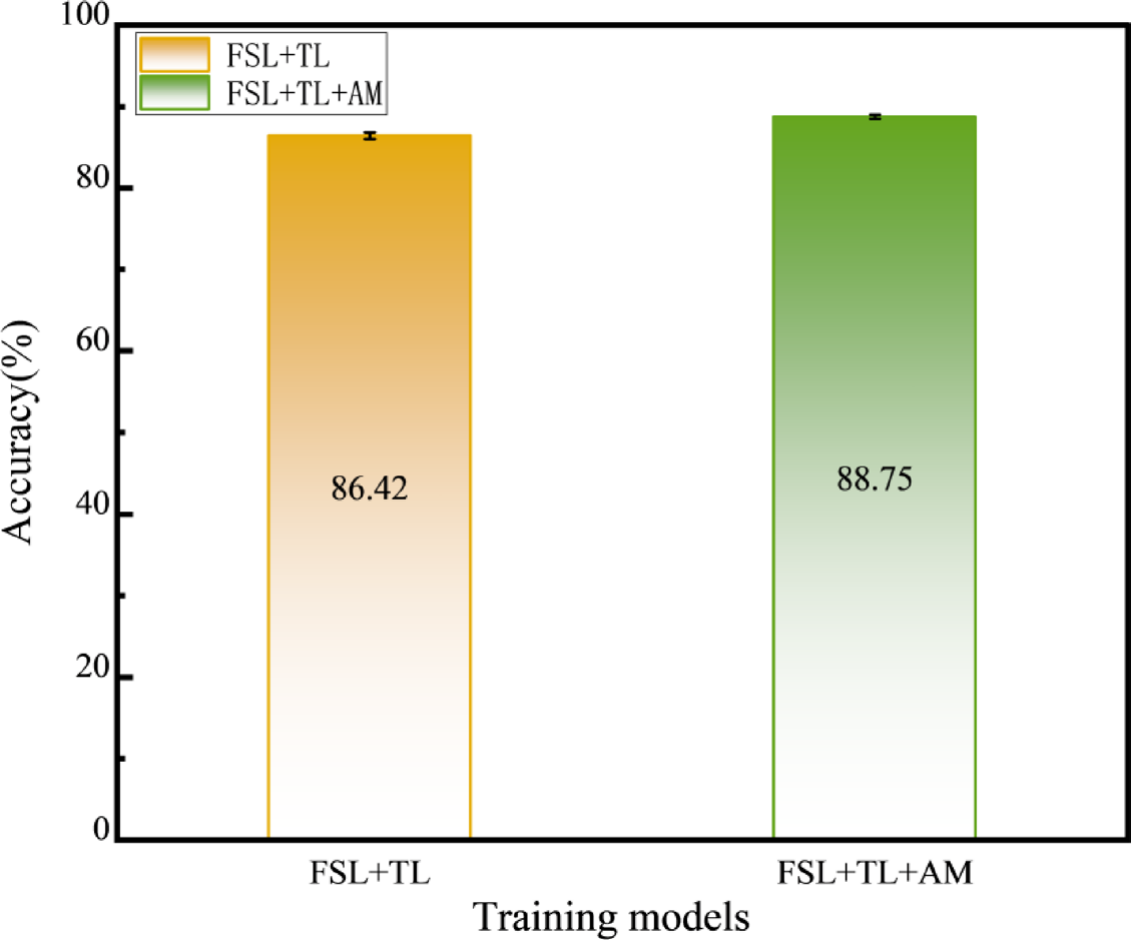
Diagnostic Results Under Imbalanced Fault Samples.

In real industrial scenarios, fault samples for certain operating conditions may accumulate over time, while other fault categories remain few-shot. This experiment evaluates model generalization and robustness under imbalanced data by fine-tuning the CWRU pre-trained model on combined TR1, TR2, and TR3 datasets, followed by testing on the mixed-condition TS1 + 2 + 3 set. Comparative analysis is conducted between FSL + TL(Siamese network pre-training + fine-tuning with limited target-domain samples) and FSL + TL + AM(FSL + TL enhanced with an attention mechanism), as shown in Fig. 8.The objective is to validate whether these methods stably improve cross-condition generalization under data imbalance and whether the attention mechanism mitigates inner/outer race fault misclassification. Diagnostic results are shown in Fig. 9.

**Fig. 10:**
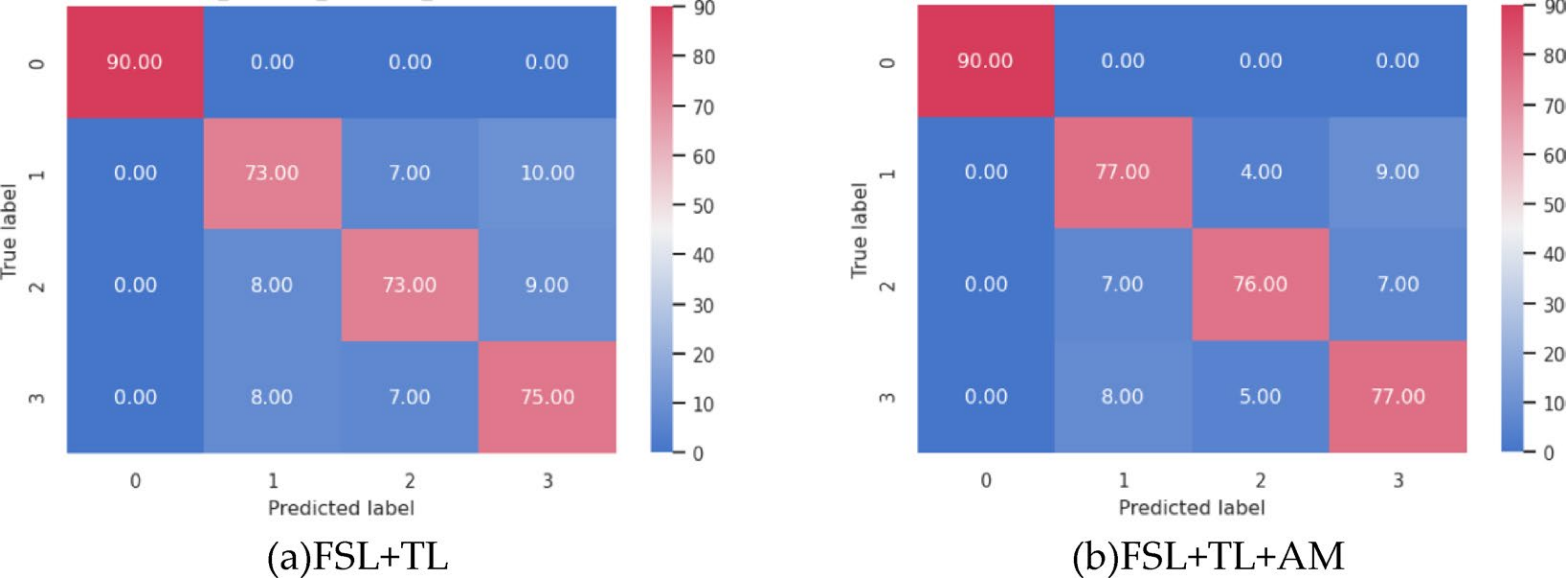
Confusion Matrices of Diagnostic Results.

Under mixed conditions, the FSL + TL method achieves an overall accuracy of 86.42%, while the FSL + TL + AM method improves accuracy to 88.75%—a 2.33% enhancement, primarily due to the attention mechanism (AM) dynamically enhancing discriminative features (e.g., impact harmonics) for inner/outer race faults. Figure 10 presents confusion matrices for both methods using 360 test samples under mixed conditions, while Table 5 quantifies their evaluation metrics per fault class. Although residual confusion persists between inner and outer race faults due to limited samples in specific target-domain conditions, both methods achieve high performance. Notably, the attention mechanism critically improves discrimination: the inner race F1-score increases from 0.82 (FSL + TL) to 0.85 (FSL + TL + AM), and the outer race F1-score rises from 0.82 to 0.84. AM effectively addresses data imbalance by prioritizing fault-sensitive frequency bands, demonstrating robustness in complex industrial scenarios.

**Table 5:** Evaluation metrics of the two methods.

Method	Index	0	1	2	3	Macro-avg
FSL + TL	Precision(%)	100	82.02	83.91	79.79	86.43
	Recall(%)	100	81.11	81.11	83.33	86.39
	F1-Score	1.00	0.82	0.82	0.82	0.86
FSL + TL + AM	Precision(%)	100	83.70	89.41	82.80	89.98
	Recall(%)	100	85.56	84.44	85.56	89.14
	F1-Score	1.00	0.85	0.87	0.84	0.89

**Fig. 11:**
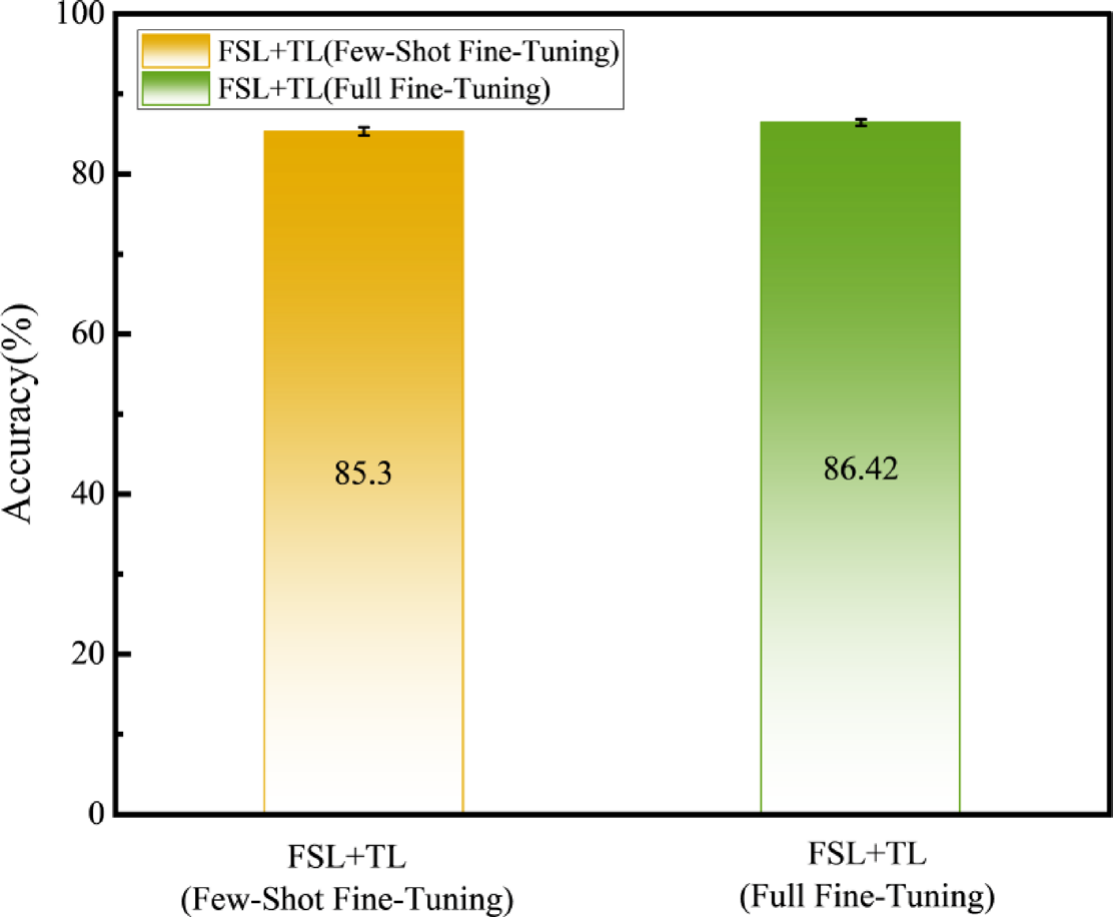
Impact of Fine-Tuning Sample Size on Accuracy.

Furthermore, our analysis reveals that the accuracy of the FSL + TL method improves with increasing fine-tuning sample size, as shown in Fig. 11. The accuracy under target-domain few-shot fine-tuning reaches 85.30%, which increases to 86.42% (a 1.12% improvement) when fine-tuned with additional samples. More target-domain data reduces overfitting by providing richer feature distributions for parameter optimization, thereby boosting generalization on unseen industrial scenarios.

**Fig. 12:**
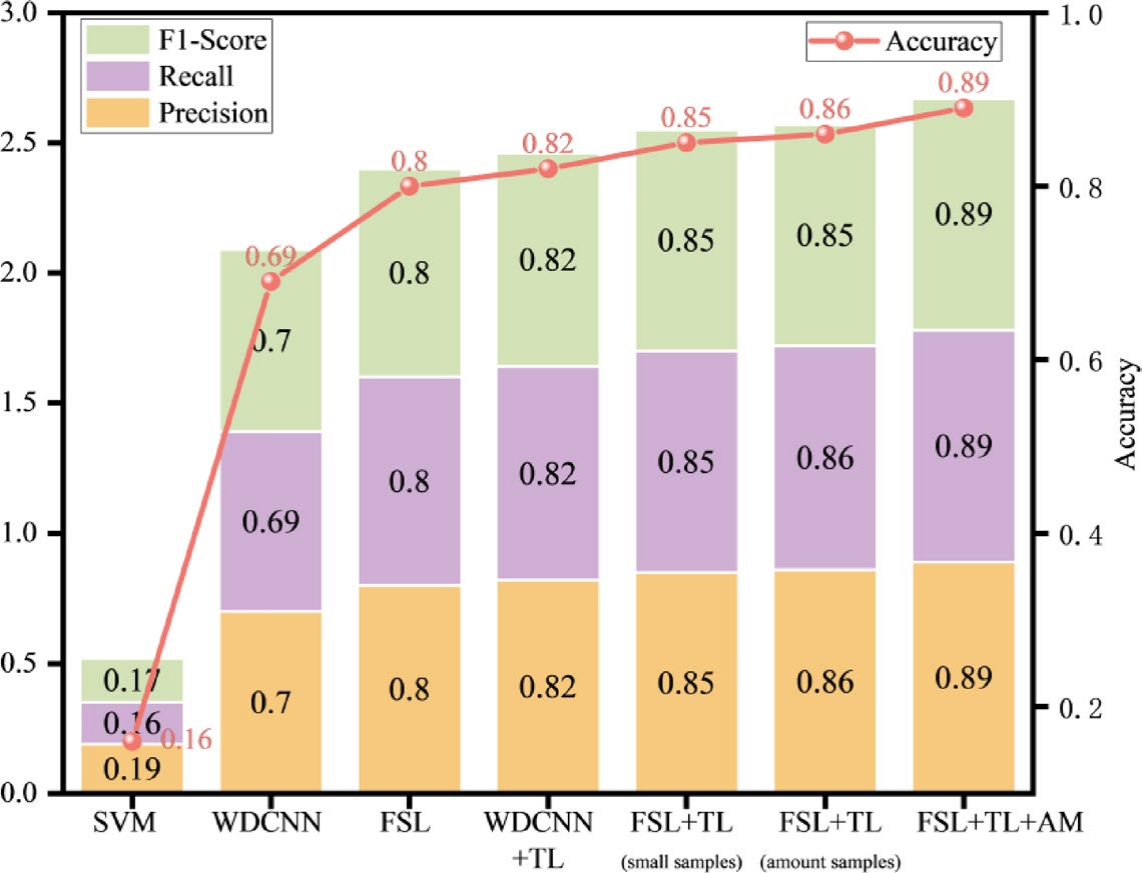
Macro-Average Metrics and Accuracy Comparison.

Finally, a comprehensive analysis of macro-averaged evaluation metrics and model accuracy (Fig. 12) demonstrates that the proposed FSL + TL method, through the integration of transfer learning and few-shot strategies, significantly enhances model robustness and generalization capability. The incorporation of the attention mechanism further optimizes critical feature extraction, markedly improving the model’s generalization capacity and classification stability for complex fault patterns.

### Analysis of FSL + TL + AM’s computational efficiency

To substantiate our claims regarding real-time and low-computational demands, we conducted a detailed analysis of model parameters, computational complexity, and inference efficiency. The benchmark comparison results are summarized in Table 6. Our FSL + TL + AM framework achieves efficient industrial deployment through the following optimizations:

1. Parameter Efficiency: The proposed framework achieves a compact parameter size of 1.49 M, significantly lower than conventional meta-learning model, through two key design choices: the Siamese-WDCNN architecture employs wide convolutional kernels (64 × 1 stride) to retain low-frequency fault features with reduced layer depth, and selective parameter fine-tuning updates only 35% of parameters (fully connected and attention layers) during transfer learning, minimizing memory overhead while preserving pre-trained knowledge.
2. FLOPs Optimization: With 3.68G FLOPs per inference, computational efficiency is ensured via attention-guided feature pruning and hardware-aware layer fusion, cutting memory access latency. These optimizations balance accuracy and computational load for edge devices.
3. Inference Speed: Tested on an NVIDIA RTX 3060 (PyTorch 1.40), the model processes samples in 14.08 ms (excluding data loading), meeting stringent industrial real-time requirements. This efficiency stems from architectural lightweighting and deployment optimizations.

**Table 6:** Benchmark comparisons.

Component	Parameters(M)	FLOPs(G)	Inference Time (ms)
Siamese-WDCNN	0.94	2.86	11.09
+ TL	+ 0.32	+ 0.44	+ 1.12
+ AM	+ 0.23	+ 0.38	+ 1.87
**Total**	**1.49**	**3.68**	**14.08**

These results confirm that FSL + TL + AM balances accuracy and efficiency, making it deployable on edge devices for real-time hydrodynamic transmission monitoring.

## Conclusions

This study investigates the FSL + TL method for bearing fault diagnosis in high-power hydrodynamic transmissions, experimentally validating its efficacy and generalization capability under few-shot conditions. The methodology first employs a Siamese Wide Convolutional Neural Network (Siamese-WDCNN) integrated with few-shot learning for pre-training on public bearing datasets to extract essential features of vibration signals. Subsequently, a transfer learning strategy is introduced to adapt the pre-trained model to hydrodynamic transmission testbed data, combined with limited target-domain samples for fine-tuning, thereby enhancing cross-condition diagnostic performance.

Experimental results demonstrate that the FSL + TL method outperforms conventional SVM, WDCNN, FSL, and WDCNN + TL approaches under both cold-start and imbalanced fault sample conditions, achieving an accuracy of 85.30% in mixed operating conditions. Further incorporation of an attention mechanism elevates the accuracy to 88.75% (FSL + TL + AM), effectively improving the generalization capability of the bearing fault diagnosis model. By synergistically leveraging the strengths of few-shot learning and transfer learning, the proposed method achieves efficient bearing fault diagnosis under data scarcity and complex operating conditions, exploring a viable pathway for industrial equipment health monitoring.

While the current work exhibits limitations in model adaptability and computational efficiency, future research will focus on developing lightweight architectures and integrating multi-source information fusion to enhance diagnostic stability and generalization.

## Data Availability

The datasets used and/or analysed during the current study available from the corresponding author on reasonable request.
